# A Systematic Review and Meta-Analysis of Prophylactic Vasopressors for the Prevention of Peri-Intubation Hypotension

**DOI:** 10.3390/diseases13010005

**Published:** 2024-12-31

**Authors:** Hollie Saunders, Subekshya Khadka, Rabi Shrestha, Hassan Z. Baig, Scott A. Helgeson

**Affiliations:** Department of Pulmonary and Critical Care, Mayo Clinic, Jacksonville, FL 32224, USA; khadka.subekshya@mayo.edu (S.K.); shrestha.rabi@mayo.edu (R.S.);

**Keywords:** intubation, hypotension, vasopressors

## Abstract

Background/Objectives: Peri-intubation hypotension is a known complication of endotracheal intubation. In the hospital setting, peri-intubation hypotension has been shown to increase hospital mortality and length of stay. The use of prophylactic vasopressors at the time of sedation induction to prevent peri-intubation hypotension has been raised. This systematic review and meta-analysis aims to review the safety and efficacy of this practice. Methods: The study was fully registered with PROSPERO on 13 October 2022, and screening for eligibility was initiated on 20 September 2024. Randomized controlled trials, along with retrospective or prospective cohort studies, were included in the search. The terms “peri-intubation hypotension”, “vasopressors”, “intubation”, and “anesthesia induced hypotension” were used to search the title/summary in PubMed, Cochrane Library, and Google Scholar databases. An assessment of bias for each study was conducted using the Newcastle-Ottawa Quality Assessment Scale. The primary outcome was the rate of hypotension peri-intubation. Any complications secondary to hypotension or vasopressors were the secondary outcome. Results: We identified 13 studies, which were all randomized controlled studies, to include in the final analysis. The risk ratio for preventing peri-intubation hypotension was 1.6 (95% CI, 1.2–2.14) with the use of prophylactic phenylephrine while giving propofol versus no prophylactic vasopressors and 1.28 (95% CI 1.03–1.60) with the use of ephedrine. Conclusions: These findings suggest that in patients undergoing intubation in the operating room with propofol, prophylactic vasopressors given with induction for intubation decrease the odds of hypotension.

## 1. Introduction

The need for endotracheal (ET) intubation spans from emergent placement for respiratory failure to routine intubation for a surgical procedure requiring anesthesia. The medical specialties often faced with the need to intubate include anesthesia, emergency medicine, and intensive care medicine. One of the largest risks of ET intubation is hypotension or cardiovascular collapse. The true incidence of peri-intubation hypotension (PIH) is difficult to assess due to a lack of formal diagnostic criteria, whether a hemodynamic or time duration cut-off. In the intensive care unit (ICU) setting, the incidence of PIH is estimated to be around 20–52%, with 2.7–3.1% complicated by cardiac arrest [1,2]. One study found a 4% incidence of PIH in the operating room as compared to 28% in the ICU [3]. Other studies looking at the operating room reported rates of PIH ranging from 9–60% with general anesthesia induction [4,5,6,7]. The proposed causes of PIH include increased thoracic pressure when introducing positive pressure ventilation resulting in decreased venous return to the heart, pre-existing volume depletion, the loss of adrenergic tone following sedation, or direct effects of sedative agents used at the time of intubation on the cardiovascular system [8,9]. Any period of hypotension increases morbidity from the possible ischemia of vital organs [10,11,12].

The prevention of PIH has been a topic of interest across all subspecialties involved. In the hospital setting, PIH has been shown to have an association with increased patient mortality. Even when adjusting for baseline characteristics, multiple studies have demonstrated a significant association between PIH and ICU, hospital, and 28-day mortality [1,13]. Methods to prevent PIH or cardiovascular collapse have included investigations into patient-related factors, such as age and pre-existing co-morbidities, as well as a comparison of medications used for sedation and/or paralysis [13,14,15]. One study introduced the use of a 10-step care bundle to help decrease ET intubation-related complications, including PIH [16]. One aspect of this bundle was the early initiation of norepinephrine if the diastolic blood pressure remained < 35 mmHg. Current guidelines do not suggest the use of prophylactic vasopressors at the time of intubation to prevent PIH. Instead, the practice focuses on the treatment of hypotension once it occurs, despite known associated mortality. However, the practice of prophylactic vasopressors has been adopted by some providers based on experience [17]. Due to their easy availability as a “push” dose, the frequently encountered vasopressors in these instances include ephedrine and phenylephrine [17].

Given the complication rate associated with intubation and outcomes, further evaluation into safer intubation practices is warranted. This analysis sought to review both the effectiveness and safety of peri-intubation hypotension prevention with prophylactic vasopressors.

## 2. Materials and Methods

### 2.1. Information Sources and Search Strategy

The Preferred Reporting Items for Systematic Reviews and Meta-Analysis statements were followed for this study [18]. The study was fully registered with PROSPERO (CRD42022364361) on 13 October 2022, and no other ongoing studies addressing this question were noted. A protocol was not prepared and cannot be accessed. The manuscript was formatted with adherence to the PRISMA checklist.

A literature review was performed following registration on 20 September 2024, with electronic searches of Ovid MEDLINE/PubMed, Google Scholar, and the Cochrane Library. Randomized controlled trials, along with retrospective or prospective cohort studies, were included in the search.

The terms “vasopressors”, “intubation”, “peri-intubation hypotension”, “anesthesia induced Hypotension”, and “hypotension” were used to search the title/summary in PubMed, Cochrane Library, and Google Scholar databases. The bibliographies of identified studies were also reviewed to identify additional studies that had not been identified by the initial searches. The full search strategy is available to view in the supplemental section as Supplemental Figure S1. Two investigators independently reviewed the final literature search and extracted data.

### 2.2. Eligibility Criteria and the Selection Process

We applied the following inclusion criteria based on abstract information: original studies, studies on prevention of peri-intubation hypotension, investigation was either randomized, retrospective, or observational, but had comparator groups, and articles that reported rates of hypotension with and without intervention. Articles were limited to those in English. If studies did not meet the defined criteria on the initial screen of abstract and title screening, they were excluded. Those included after this initial assessment were then assessed with the following criteria: studies measuring hemodynamic parameters at the time of intubation or induction of anesthesia, intubation performed within hospital settings (ED, ICU, wards, OR), studies with the use of any vasopressor used before or during induction before the onset of hypotension, and all patients aged > 18 years. If these criteria were met, then the study was included in the analysis. We excluded studies with no specification of the values used to estimate the frequency of peri-intubation hypotension, as well as studies that used vasopressors to treat rather than prevent PIH once it occurred. Studies that were identified as being performed on pediatric (age < 18 years old) and obstetric populations were excluded.

### 2.3. Data Collection Process

A database was created using all selected papers and the following variables: scenario of intubation, number of patients in control and intervention group, type of study, type of drug intervention in the treatment group, route of drug administration, complications at the time of intubation, hypotension events during intubation, and patient demographics (age, gender, and race). All data pertaining to these outcomes were independently extracted from the published manuscript by two reviewers (S.K. and R.S.) and in the case of disagreements, other reviewers (H.S. and S.H.) were used. There was no contact with authors from the selected studies for additional data.

### 2.4. Risk of Bias Assessment

An assessment of bias for each study, using the Newcastle-Ottawa Quality Assessment Scale, was performed with results in Supplemental Table S1 [19]. Two reviewers (S.H. and H.S.) independently evaluated the included studies’ quality with the appraisal tool. In the case of disagreement, a third reviewer (S.K.) was used. A good-quality study was defined as having 3 or 4 stars in the selection domain, 1 or 2 stars in the comparability domain, and 2 or 3 stars in the outcomes domain. A poor-quality study was defined as having 0 or 1 star in the selection domain, 0 stars in the comparability domain, or 0 or 1 star in the outcome domain. Funnel plots were generated to demonstrate publication bias with effectiveness (proportion) against precision (standard error).

The primary outcome was the rate of hypotension peri-intubation. The secondary outcome was complication rates secondary to the hypotension or vasopressors. The definition of PIH for each study was detailed in Table 1.

### 2.5. Synthesis Methods

A Cohen’s kappa score was calculated for inter-rater agreement testing. Peri-intubation hypotension odds ratios with 95% confidence intervals (CIs) were analyzed by a DerSimonian and Laird random effects model using Revman 5^®^ Software v5.4.1 (London, UK). A risk ratio was obtained for the risk of achieving normotension during intubation. Heterogeneity was considered to be high, moderate, or low and was quantified with an I2 of >75%, >50%, and >25%, respectively.

## 3. Results

**Figure 1:**
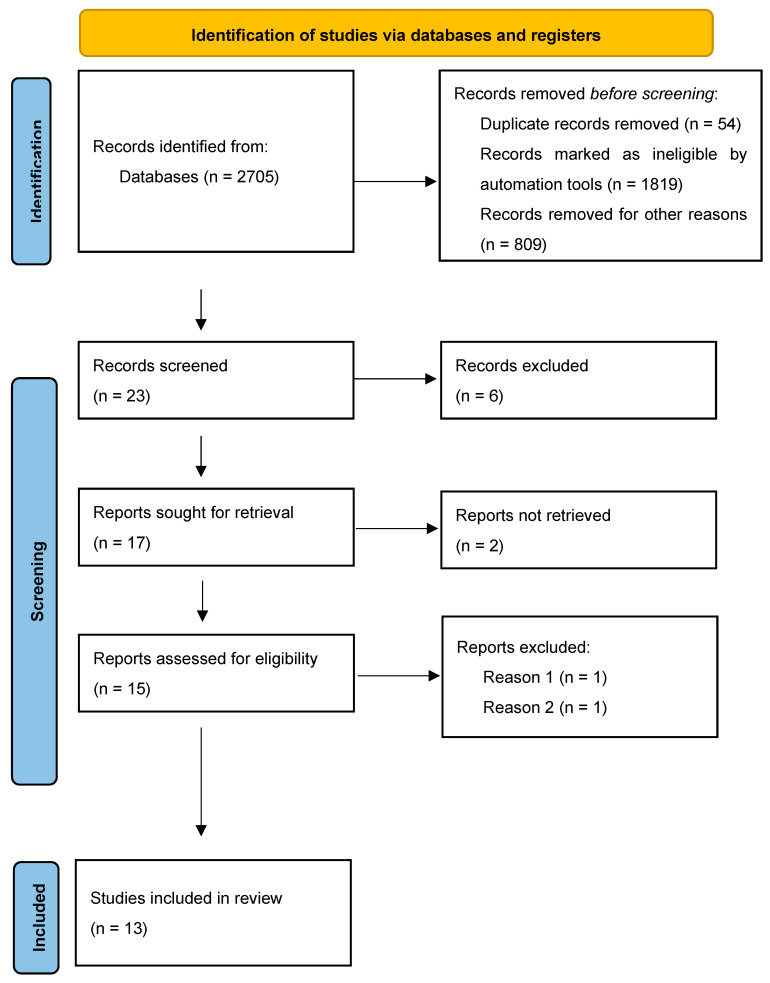
Flow chart of identified and selected studies.

The initial literature search yielded around 2705 citations. Figure 1 demonstrates the flow of selected studies. A total of 23 articles were identified based on the title and abstract pertaining to the desired topic [1,17,20,21,22,23,24,25,26,27,28,29,30,31,32,33,34,35,36,37,38,39,40]. Six studies were removed before selection based on defined criteria as stated above [17,36,37,38,39,40]. Full articles from 17 studies were retrieved with 2 excluded [1,35] for various reasons (i.e., lack of control measure, study type). Following retrieval, an additional two studies were removed. Reason 1 (noted in Figure 1) for removal was a lack of numerical value of the rate of hypotension in both the control and intervention groups. The two groups were compared graphically and favored intervention but could not be included in the analysis [34]. Reason 2 for exclusion was a retrospective study design with a reactive approach for the intervention (i.e., hypotension occurred and was then treated) as opposed to a preventative approach [33].

We identified 13 studies, which were all randomized controlled studies, to include in the final analysis. The inter-rater agreement between the two review authors was 100% when selecting studies for the primary outcome with Cohen’s kappa of 1.0 [20,21,22,23,24,25,26,27,28,29,30,31,32]. Three studies investigated the use of phenylephrine [21,22,24], eight investigated the use of ephedrine [23,25,26,28,29,30,31,32], and two studies investigated both phenylephrine and ephedrine [20,27]. Table 1 demonstrates the study characteristics of the included studies. The sedating agent was propofol for all of the studies. The studies spanned from 1995–2023. All the studies were performed in the operating room with no description of the type of procedure or who performed the sedation and intubation.

The patient characteristics, per the study, are displayed in Table 2. A total of 1358 patients were included with 817 (60%) receiving vasopressors. The median age was 37.2 years (IQR 34.5–63.4) with 53.9% female for the combined cohort of patients. If the pre-intubation mean arterial pressure (MAP) and heart rate were described, these were reported in Table 2.

**Figure 2:**
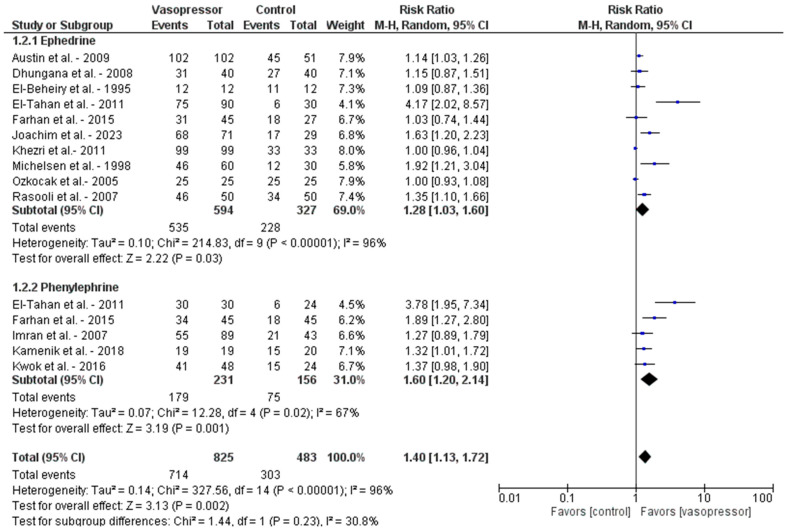
Forest Plot of studies addressing the use of vasopressors to prevent peri-intubation hypotension [20,21,22,23,24,25,26,27,28,29,30,31,32].

The risk ratio of maintaining normotension with both phenylephrine and ephedrine, when used prophylactically, was 1.40 (95% CI, 1.13–1.72). The risk ratio of preventing peri-intubation hypotension was 1.6 (95% CI, 1.2–2.14) with the use of prophylactic phenylephrine while giving propofol versus no prophylactic vasopressors and 1.28 (95% CI 1.03–1.60) with the use of ephedrine versus no prophylactic vasopressors. The Forest plot is displayed in Figure 2.

Complications from prophylactic phenylephrine occurred in 7/201 (3.5%) of patients, with bradycardia being the most common complication (n = 4). Kwok et al. [21] reported two (4.2%) cases of bradycardia requiring treatment with atropine, Kamenik et al. [22] reported three (14.3%) cases of hypertension causing medication to be held, and two (9.5%) cases of bradycardia requiring treatment with atropine. Imran et al. [24] and Farhan et al. [20] did not report any complications of bradycardia or hypertension. There was no mention of these bradycardic episodes causing any other complications. In the studies using ephedrine, five studies reported a significant increase in the heart rate in the treatment groups; none of these studies reported the need for intervention or other adverse effects [23,27,28,30,32].

**Figure 3:**
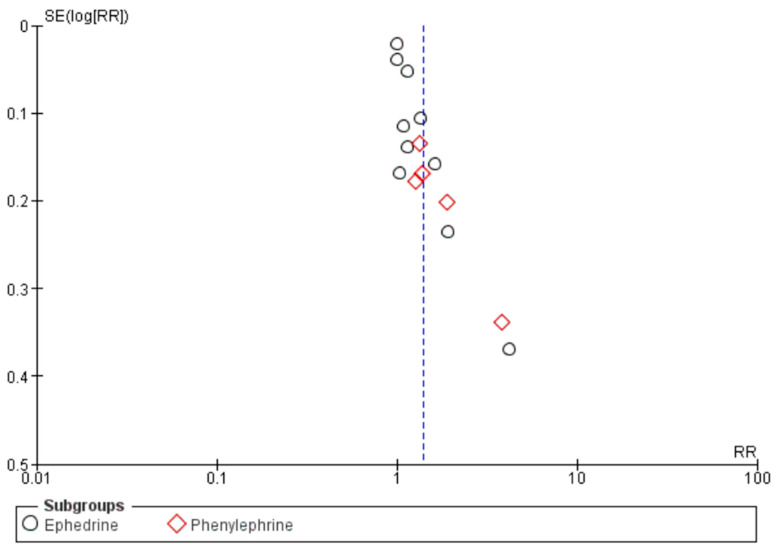
Funnel plot of included studies with effectiveness (proportion) against precision (standard error).

Based on the funnel plot for the primary outcome, there was no significant imbalance for phenylephrine or ephedrine (Figure 3). These results suggest no significant publication bias in this meta-analysis but because of the limited number of studies, undetected bias may still be present.

## 4. Discussion

This scoping study was conducted to summarize the available literature qualitatively and quantitatively on the use of vasopressors during the peri-intubation period to prevent PIH. We found a consistent association of vasopressors with higher rates of maintaining blood pressure from a meta-analysis of 13 studies. There were minimal complications from the use of vasopressors. This finding supports the hypothesis that the prophylactic use of vasopressors reduced the rate of peri-intubation hypotension.

This may have implications across all ET intubating specialties including intensive care, anesthesia, and emergency care. There are no current studies looking at surgical outcomes or long-term outcomes when PIH occurs in the OR. The only literature for outcomes and PIH has been in the emergency department and intensive care unit. Despite this, the mechanism by which PIH occurs is likely to be the same across all intubating settings, as well as the physiological impact of this. As previously noted, even when adjusting for baseline characteristics, studies have demonstrated a significant association between PIH and ICU and hospital mortality and ICU and hospital length of stay [1,13]. For example, Heffner at al. reported a hospital mortality of 33% in those who experienced PIH versus a mortality of 21% in those who did not [13]. This was similar to the ICU mortality findings by Russotto et al., which was found to be 40.7% in those who experienced PIH versus 26.3% in those who did not [1]. Some predictive factors for PIH have been identified, most often related to patient-related factors such as age and blood pressure prior to intubation [13,14]. For these reasons, some practitioners across all intubating settings already employ the use of prophylactic vasopressors to mitigate PIH based on current knowledge and personal experience [17]. The wisdom of this practice has been questioned, especially with a recent review article finding no difference in improvement in post-intubation hypotension with pre-intubation vasopressors [41]. This study examined critically ill patients mostly intubated in the emergency department or intensive care unit and not the operating setting, like this present study. Also, they did not compare different types of vasopressors. The review did raise good points that a common definition of post-intubation hypotension should be established and further studies on types of vasopressors, route of administration, and timing should be further studied. Another recent study performed a post-hoc analysis on two randomized controlled studies and also found no statistical difference in hypotension when vasopressors were used [42]. This study used propensity matching to minimize the effect of patient differences, but this study was limited to only having the ability to analyze the use of vasopressor infusion and not push dose, such as what was included in this current study. This difference should be further studied.

However, care must also be taken when selecting the intervention agent to prevent PIH. A known side effect of phenylephrine is reflex bradycardia due to its mechanism of action being alpha-adrenergic agonism with no action on beta-adrenergic receptors. A study assessing the incidence of adverse effects associated with “push-dose” phenylephrine in the ICU (both hypertension and bradycardia) found it to be <5% [43]. This is a low incidence but it is not negligible as reflex bradycardia can be significant enough to result in cardiac arrest [43]. In our analysis, this incidence was found to be slightly lower, in 3.5% of patients who received phenylephrine. Only 1.9% required intervention for this and none progressed to cardiac arrest. This would indicate the safety of this practice but highlights the need to be aware of potential adverse effects so that they can be addressed promptly. Furthermore, it can be argued that the maintenance of cardiac output, rather than MAP, is more important in this patient population. The reflex bradycardia caused by phenylephrine may adversely result in reduced cardiac output, despite the increase in MAP, as cardiac output is dictated by a combination of stroke volume and heart rate. In contrast to phenylephrine, ephedrine is a sympathomimetic with alpha- and beta-adrenergic receptor activity, meaning that an increase in heart rate and cardiac output can be seen. Possible adverse effects of ephedrine are tachycardia and arrhythmias; this can be of concern in the elderly population or those with existing heart disease [26]. Despite this, Michelsen et al., who studied a more elderly female population reported a significant increase in heart rate in the treatment groups but did not report any adverse events related to this [30]. Specific patient characteristics that may make them vulnerable to the adverse effects of push-dose vasopressors have not been specifically identified.

Further assessment of patient selection and sedative agents can be performed to help guide this practice. In all the studies included, the sedative agent used was propofol. Propofol demonstrates a dose-dependent effect on the cardiovascular system, including a reduction in systemic vascular resistance through decreasing sympathetic tone, as well as inhibition of the physiological baroreceptor response [44]. This can be more pronounced in the elderly and physiologically compromised patients, a population more frequently encountered in the ICU/ED setting [44]. For this reason, either a reduced dose of propofol or an alternative agent may be used for induction. According to the National Emergency Airway Registry, the most frequently used induction agents in the emergency department are etomidate (69%), midazolam (16%), fentanyl (6%), and ketamine (3%) [45,46]. However, even without direct cardiovascular effects, these other sedative agents have been implicated in PIH [47]. Further studies are necessary to evaluate the effectiveness of induction agents other than propofol and prophylactic measures to prevent PIH.

This presents a limitation in the current study, as the effectiveness of vasopressors to prevent PIH using alternate sedative agents was not addressed. A further limitation of the study of PIH is that there is no general consensus on the definition of PIH. Some studies noted a specific value of mean arterial pressure or systolic blood pressure, while others defined it as the need for additional vasopressors for treatment. This makes a comprehensive study of the condition and its implications challenging. Additionally, the exact dose of timing of administration of the prophylactic vasopressor were not always specified. However, as acknowledged above, differences exist in practice across the operating room and the ICU/ED regarding the choice and dose of sedative. This means that the timing and dose of prophylactic vasopressor may not be generalizable. With regard to patient outcomes, the results are limited to the presence or absence of PIH, and studies do not report on mortality or other perioperative complications such as myocardial infarction. However, the studied population was young (median age 37 years), with a generally lower ASA class; so these results may not be generalizable. Finally, all studies were performed in the operating room setting with relatively young patients and low ASA class. This means that the findings may not be able to be generalized to the ICU or ED settings where patients are generally sicker and less physiologically compensated. However, this does mean that this patient population may be more vulnerable to PIH. Further studies will be required in an ICU or ED setting to understand efficacy and safety in this patient population.

Several questions remain unanswered regarding prophylactic vasopressors with intubation. It remains to be determined which patient population would benefit the most from this practice. As there has previously been a study on patient factors that increase the risk of PIH, such as age, co-morbidities, and blood pressure before intubation, it may be valuable to understand whether the application of vasopressors to prevent PIH may be of more impact in this population. There was not enough detail in the included studies to shed any light on this question. Future studies on different patient populations (i.e., type of surgery, different anesthetics, or different ASA classes) would be necessary to explore this question. There is potential to expand the practice of prophylactic vasopressors during intubation induction to the intensive care unit or emergency department patients to prevent PIH.5.

## 5. Conclusions

In conclusion, these findings suggest that in patients undergoing intubation in the operating room with propofol, prophylactic phenylephrine or ephedrine given with induction for intubation decreases the odds of hypotension. Future studies are necessary to determine the ideal patient population to use this practice and whether this can be extrapolated to the emergency department and intensive care unit.

## Figures and Tables

**Table 1:** Study characteristics of articles included in meta-analysis [20,21,22,23,24,25,26,27,28,29,30,31,32]. (RCT—randomized control trial, OR—operating room, MAP—mean arterial pressure, SBP—systolic blood pressure, DBP—diastolic blood pressure).

Study	Design	Location	Intervention	Sedation Agent	Control (n)	Intervention (n)	Definition of Hypotension
Austin et al., 2009	RCT	OR	Ephedrine	Propofol	51	105	Requiring treatment
Dhungana et al., 2008	RCT	OR	Ephedrine	Propofol	80	40	20% decrease in SBP from baseline
El-Beheiry et al., 1995	RCT	OR	Ephedrine	Propofol	24	12	SBP decrease from baseline
El-Tahan et al., 2011	RCT	OR	Ephedrine Phenylephrine	Propofol	30	90 30	20% decrease in MAP from baseline
Farhan et al., 2015	RCT	OR	Ephedrine Phenylephrine	Propofol	45	45 45	20% decrease in MAP from baseline
Joachim et al., 2023	RCT	OR	Ephedrine	Propofol	29	58	SBP < 80 mmHg or >20% decrease from baseline
Khezri et al., 2011	RCT	OR	Ephedrine	Propofol	66	99	Comparison of change in MAP
Michelsen et al., 1998	RCT	OR	Ephedrine	Propfol	30	60	SBP < 80 mmHg
Ozkocak et al., 2005	RCT	OR	Ephedrine	Propofol	50	25	Comparison of change in SBP, DBP
Rasooli et al., 2007	RCT	OR	Ephedrine	Propofol	50	50	20% decrease from baseline
Imran et al., 2007	RCT	OR	Phenylephrine	Propofol	43	89	20% decrease in SBP from baseline
Kamenik et al., 2018	RCT	OR	Phenylephrine	Propofol	19	21	Requiring additional boluses of phenylephrine
Kwok et al., 2016	RCT	OR	Phenylephrine	Propofol	24	48	SBP < 80 mmHg

**Table 2:** Baseline patient characteristics in each included study [20,21,22,23,24,25,26,27,28,29,30,31,32]. (MAP—mean arterial pressure, HR—heart rate, bpm—beats per minute, ASA—American Society of Anesthesiologists, NR—not reported).

Study	N	Female (%)	Age (Years)	Weight (kg)	MAP (mmHg)	HR (bpm)	ASA Class Included
Austin et al., 2009	156	59.6	47.4	78.8	NR	NR	I, II, III
Dhungana et al., 2008	120	77.5	35.2	50.0	96.2	81.3	I, II
El-Beheiry et al., 1995	36	55.6	31	72.9	NR	81.3	I, II
El-Tahan et al., 2011	150	27	37.2	77.3	90	78	III, IV
Farhan et al., 2015	135	51.8	34.9	64.5	92.8	83.2	I, II
Joachim et al., 2023	87	37.8	36.1	58.4	90.8	83.8	I, II
Khezri et al., 2011	165	52	31.6	73.4	NR	NR	I, II
Michelsen et al., 1998	90	100	68	65	102.5	81	I, II, III
Ozkocak et al., 2005	75	48	58.7	72.2	87	82	I, II
Rasooli et al., 2007	100	48	68.8	62.3	117.7	NR	I, II
Imran et al., 2007	132	47	34.0	64.6	96.3	85.6	I, II
Kamenik et al., 2018	40	29.5	64.4	78.3	102	69	I, II, III
Kwok et al., 2016	72	66.7	62.3	59.0	112.5	76.6	I, II

## Data Availability

Data are available with a reasonable request.

## Supplementary Materials

The following supporting information can be downloaded at https://www.mdpi.com/article/10.3390/diseases13010005/s1: Figure S1: Full search strategy; Table S1: Newcastle-Ottowa Quality assessment scale.
