# Supplementary material for: A Systematic Review and Meta-Analysis of Prophylactic Vasopressors for the Prevention of Peri-Intubation Hypotension
Source: Diseases. 2024 Dec 31;13(1):5. doi: 10.3390/diseases13010005 (PMC11764260; doi:10.3390/diseases13010005)
Supplement: Supplementary file 1 [file diseases-13-00005-s001.zip › diseases-3269300-supplementary.pdf]

Table S1:

Newcastle-Ottowa Quality assessment scale.

Study	Selection (max 4 stars)				Comparability (max 2 stars)	Outcome (max 3 stars)			Overall Quality
	S-1	S-2	S-3	S-4	C-1	O-1	O-2	O3	
**Austin et al. 2009**	*	*	*	*		*	*	*	Moderate
**Dhungana et al. 2008**	*	*	*	*		*	*	*	Moderate
**El-Beheiry et al. 1995**	*	*	*	*		*	*	*	Moderate
**El-Tahan et al. 2011**	*	*	*	*		*	*	*	Moderate
**Farhan et al. 2015**	*	*	*	*		*	*	*	Moderate
**Joachim et al. 2023**	*	*	*	*		*	*	*	Moderate
**Khezri et al. 2011**	*	*	*	*		*	*	*	Moderate
**Michelsen et al. 1998**	*	*	*	*		*	*	*	Moderate
**Ozkocak et al. 2005**	*	*	*	*		*	*	*	Moderate
**Rasooli et al. 2007**	*	*	*	*		*	*	*	Moderate
**Imran et al. 2007**	*	*	*	*		*	*	*	Moderate
**Kamenik et al. 2018**	*	*	*	*		*	*	*	Moderate
**Kwok et al. 2016**	*	*	*	*		*	*	*	Moderate

S-1: Representativeness of the exposed cohort
S-2: Selection of the non-exposed cohort
S-3: Ascertainment of exposure
S-4: Demonstration that outcome was not present at start of study
C-1: Comparability of cohorts on the basis of the design or analysis
O-1: Assessment of outcome
O-2: Was follow-up long enough for outcomes to occur
O-3: Adequacy of follow-up of cohorts

Figure S1:

Full search strategy

Pubmed

Intubation AND vasopressors OR anesthesia induced hypotension OR peri-intubation hypotension (2,074)

Limit: Clinical trial, metanalysis, randomized control trial, systematic review (396)

Limit: 1993-2023 (406)

Filter: Humans (399)

Filter: Adults (216)

Filter: English (196)

Cochrane Library

Intubation AND vasopressors OR anesthesia induced hypotension OR peri-intubation hypotension (154)
Exclude: Pubmed (54)
Limit: 1993-2023 (54)
Filter: English (52)

Google Scholar
Peri-intubation hypotension AND vasopressors (477)
Limit: 1993-2023 (436)
